# Treatment of avulsion fracture of posterior cruciate ligament tibial insertion by minimally invasive approach in posterior medial knee

**DOI:** 10.3389/fsurg.2022.885669

**Published:** 2023-01-06

**Authors:** Huihui Guo, Yao Zhao, Liang Gao, Chen Wang, Xianbo Shang, Haitao Fan, Wendan Cheng, Chang Liu

**Affiliations:** ^1^ Fuyang People's Hospital, Fuyang, China; ^2^ Department of Orthopedics, The Second Affifiliated Hospital of Anhui Medical University, Hefei, China; ^3^ Center for Clinical Medicine, Huatuo Institute of Medical Innovation (HTIMI), Berlin, Germany; ^4^ Anhui Medical University, Fuyang, China; ^5^ Anhui Armed Police General Hospital, Hefei, China

**Keywords:** minimally invasive, posterior cruciate ligament, avulsion fracture, clinical effects, technique

## Abstract

**Objective:**

The study aims to explore the feasibility and clinical effect of posterior minimally invasive treatment of cruciate ligament tibial avulsion fracture.

**Methods:**

Posterior knee minimally invasive approach was used to treat avulsion fracture of posterior cruciate ligament (PCL) tibia in 15 males and 11 females. The length of the incision, intraoperative blood loss, operation time, postoperative hospital stay, residual relaxation, and fracture healing time were analyzed to evaluate the curative effect, learning curve, and advantages of the new technology. Neurovascular complications were recorded. During the postoperative follow-up, the International Knee Joint Documentation Committee (IKDC), Lysholm knee joint score, and knee joint range of motion were recorded to evaluate the function.

**Results:**

All 26 patients were followed up for 18–24 months, with an average of 24.42 ± 5.00 months. The incision length was 3–6 cm, with an average of 4.04 ± 0.82 cm. The intraoperative blood loss was about 45–60 ml, with an average of 48.85 ± 5.88 ml. The operation time was 39–64 min, with an average of 52.46 ± 7.64 min. The postoperative hospital stay was 2–5 days, with an average of 2.73 ± 0.87 days. All incisions healed grade I without neurovascular injury. All fractures healed well with an average healing time of 9.46 ± 1.33 weeks (range, 8–12 weeks). The Lysholm score of the affected knee was 89–98 (mean, 94.12 ± 2.49) at 12-month follow-up. The IKDC score was 87–95 with an average of 91.85 ± 2.19, and the knee range of motion was 129–148° with an average of 137.08 ± 5.59°. The residual relaxation was 1–3 mm, with an average of 1.46 ± 0.65 mm.

**Conclusion:**

This minimally invasive method provides sufficient exposure for internal fixation of PCL tibial avulsion fractures without the surgical complications associated with traditional open surgical methods. The process is safe, less invasive, and does not require a long learning curve.

## Background

As the most complicated joint of human body, the stability of knee joint depends on the surrounding ligaments to a great extent. Posterior cruciate ligament (PCL) is the necessary ligament to maintain the stability of the knee joint, and it is also the main limiting factor to prevent excessive posterior tibial movement. Its fracture and injury will greatly affect the stability of the knee joint (1).

PCL injury accounts for 3%–44% of acute knee joint injuries and is often accompanied by other ligament injuries (2–4). The avulsion fracture of PCL is a kind of knee joint injury, which can easily lead to instability of knee joint and accelerate the long-term degeneration of knee joint. The avulsion fracture of the tibia is usually caused by high-energy injuries, which are common in motorcycle accidents (5, 6). At this time, the knee joint is in a bent position or in an overextended position. If the upper end of the tibia is subjected to violence from front to back, the tension on the PCL will easily exceed its tolerance limit, which will lead to injuries such as PCL fracture. Due to the anatomical characteristics of PCL tibial attachment points, some of them are located outside the joint cavity. When the avulsion fracture of PCL tibial insertion occurs, the fracture end is often embedded in the joint capsule and surrounding soft tissues, which makes it difficult to reduce the fracture by manipulation. If the torn pieces are not displaced, nonsurgical treatment can be recommended. At present, it is considered that the main treatment for avulsion fracture of displaced PCL tibial insertion is surgical treatment to restore PCL function and knee stability (7, 8), and avulsion fracture of PCL tibial attachment is considered an indication of surgical reduction and internal fixation (9). The main surgical treatments include open reduction and internal fixation (10–13) and arthroscopic reduction and internal fixation (14–16). Although there are many case series published on the management and outcomes of PCL avulsion fractures, no optimal surgical management has been suggested (5, 17). This paper attempts to find a simpler and minimally invasive method to treat PCL tibial avulsion fracture, which does not need to dissect the surrounding soft tissues layer by layer, and at the same time better protect the surrounding muscles and blood vessels and nerves. We intend to use this new minimally invasive approach to fix PCL tibial avulsion fracture, which has been successfully applied to 26 patients. The effectiveness, safety, and advantages of this method are analyzed by using the results measurement method of clinician's specialist evaluation and patient report.

## Clinical data

### General information

**Figure 1:**
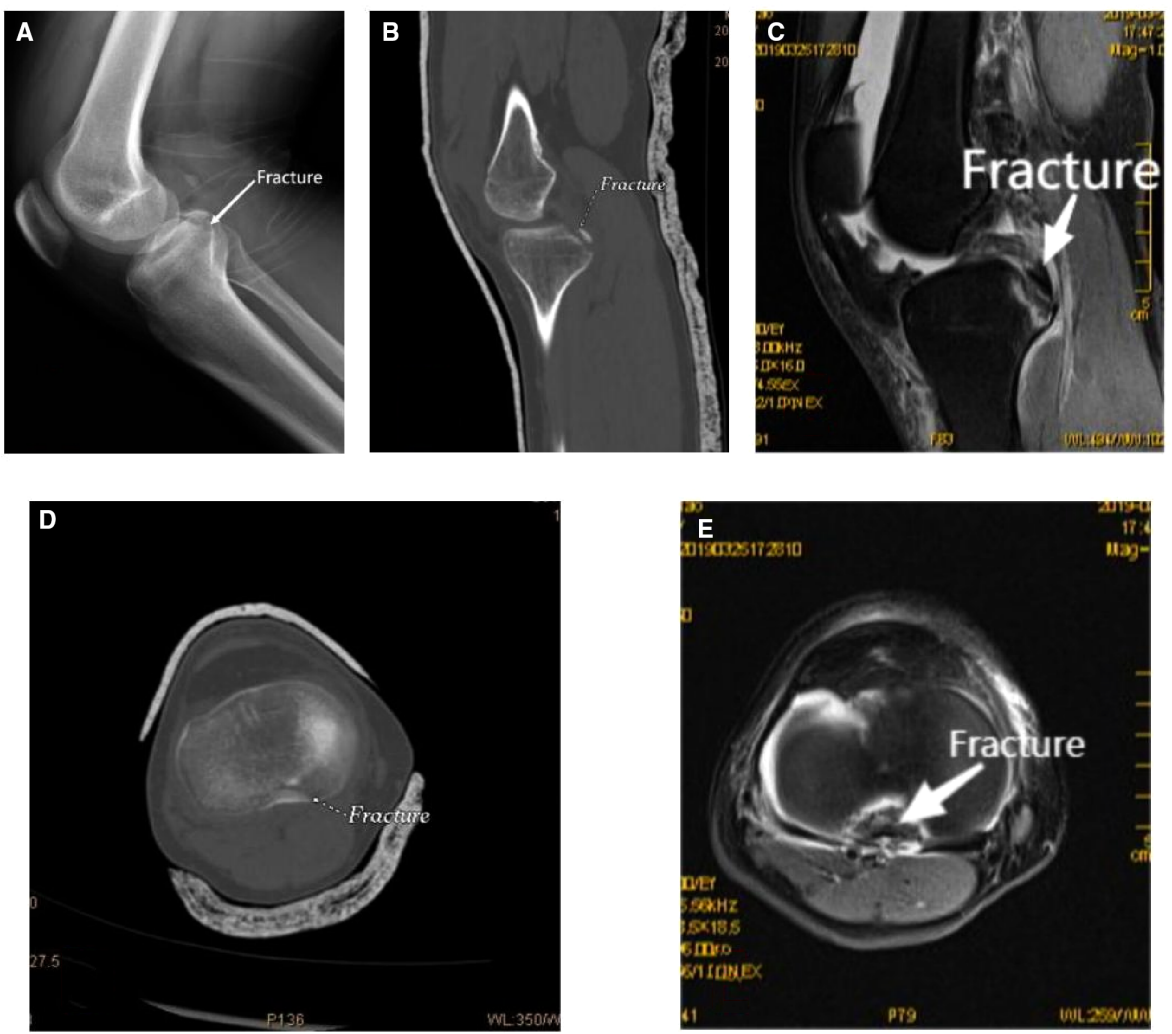
Preoperative x-ray, CT, and MRI of the patient [(**B,C**) sagittal view; (**D,E**) transverse view]. CT, computed tomography; MRI, magnetic resonance imaging.

From January 2015 to January 2020, 26 cases of PCL tibial avulsion fracture were studied retrospectively at the orthopaedics departments of both Second Affiliated Hospital of Anhui Medical University and Anhui Armed Police General Hospital, Hefei, China. After all patients were admitted to the hospital, routine biochemical and physical examinations were completed, and their physical condition and surgical tolerance were fully evaluated. Lachman test and back drawer test were positive before operation; and x-ray, computed tomography (CT) scanning + three-dimensional reconstruction, and magnetic resonance imaging (MRI) (Figure 1) examination of the knee joint of the affected limb were improved, so as to facilitate the evaluation of fracture size and the selection of internal fixation devices during operation. The consent of patients and their families was obtained before operation.

### Inclusion criteria

(1) Fresh PCL tibial avulsion fracture (fracture within 3 weeks);
(2) Lachman test and back drawer test were positive before operation;
(3) Meyers–McKeever II and III, and preoperative CT measurements of fracture block size can be fixed by hollow lag screws;
(4) Knee joint function was good before injury;
(5) Follow-up for more than 12 months, complete imaging data; and
(6) The images obtained showed that the mean fracture displacement of PCL was ≥6.7 mm (18).

### Exclusion criteria

(1) Patients with anterior cruciate ligament, collateral ligament, and meniscus injury;
(2) Preoperative MRI showed PCL rupture;
(3) Osteoarthritis with previous joint dysfunction, history of knee joint trauma or Kellgren–Lawrence grade ≥2;
(4) Patients with distal femur or proximal tibia fracture; and
(5) Preoperative surgical evaluation, patients with severe heart, respiratory, and other medical diseases who cannot be operated on.

### Surgical technique

All of the patients were operated on by three senior orthopedic surgeons of the same team. General anesthesia was used as the anesthesia method. After satisfactory anesthesia, an electric pneumatic tourniquet was tied in the prone position at the root of the thigh of the affected limb. The pressure of the tourniquet was set at 280 mmHg, and the knee joint was flexed from 30° to 45° to fully relax the medial gastrocnemius muscle.

**Figure 2:**
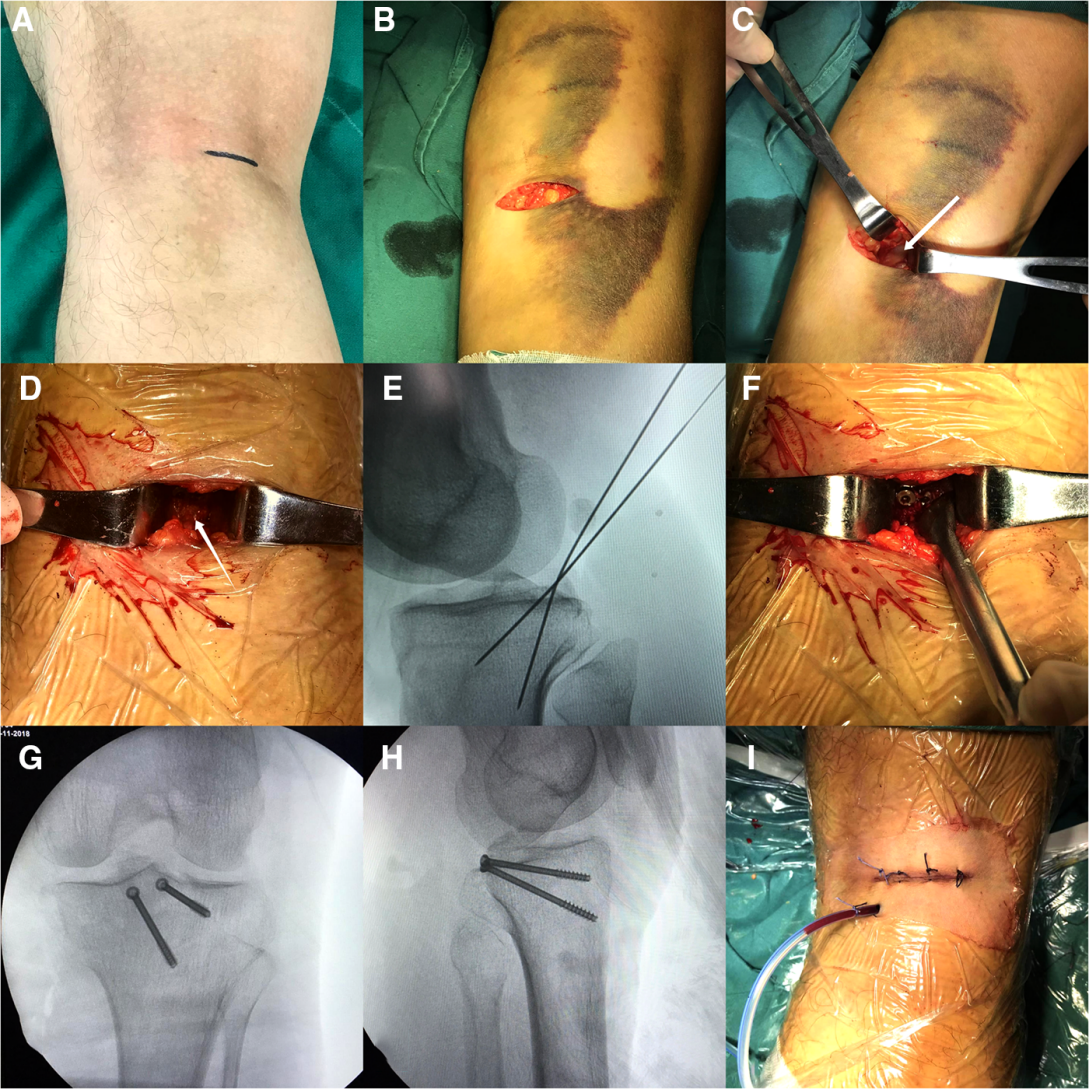
(**A**–**C**) A 3–4 cm surgical incision was performed along the preoperative marker, the gastrocnemius muscle's medial head was separated, and the joint capsule was fully exposed by pulling outward. (**D**–**F**) The joint capsule was cut open, washed, the fracture block was reduced, and the Kirschner wire was used for temporary fixation. Cannulated lag screws were used for fixation after the fracture was well positioned in fluoroscopic position. (**G**–**I**) The intraoperative fluoroscopic anterolateral x-ray film was satisfactory, and the incision was closed layer by layer after rinsing and inserting the drainage tube.

**Figure 3:**
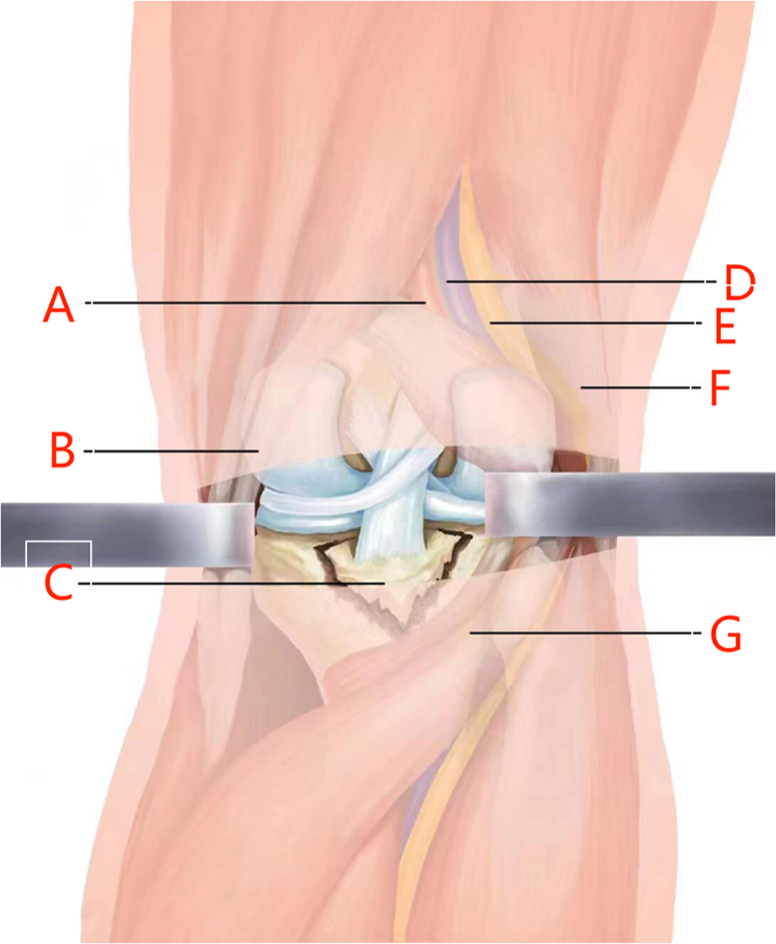
Illustration of surgical anatomy of PCL tibial avulsion fracture. [(**A**) Popliteal artery; (**B**) semitendinosus muscle; (**C**) avulsion fracture of posterior cruciate ligament; (**D**) popliteal vein; (**E**) tibial nerve; (**F**) long head of biceps femoris; (**G**) medial head of gastrocnemius muscle]. PCL, posterior cruciate ligament.

Before the operation, a 3–4 cm surgical mark was made along the medial margin of the gastrocnemius muscle from 1 to 2 cm above the popliteal stria; routine disinfection and towel laying were performed. A 3–4 cm incision was performed from the medial side of the popliteal stria along with the preoperative surgical incision mark. The medial margin of the medial head of the gastrocnemius muscle was determined. The anatomical gap between the medial head of the gastrocnemius muscle and the semitendinosus muscle was bluntly dissected with fingers, and the lateral margin of the medial head of the gastrocnemius muscle was bluntly separated from the semitendinosus muscle. The medial head of the gastrocnemius muscle and the vascular nerve in popliteal fossa were pulled outward with a thyroid hook, and the semimembrane muscle and semitendinosus muscle were pulled medially. At this time, the thick muscle belly could play a role in protecting the vascular nerve structure, and no pressure was directly applied to the vascular nerve during the operation. Therefore, dissection of the protected medial popliteal fossa is relatively safe. Until the posterior congestion and swelling of the joint capsule are exposed, the avulsion fracture fragment could be seen by a longitudinal incision of the joint capsule, and the fracture fragment was lifted along with the PCL. The surgical site was fully washed with normal saline, the surrounding soft tissue embedded in the fracture fragment was cleaned, and the fracture fragment was reduced to the bone bed. 1–2 Kirschner wires were vertically placed at the upper edge of the bone bed, and the direction and depth of the Kirschner wires were visualized to avoid damage to the surrounding articular cartilage. When the direction and position were good, 1–2 hollow lag screws with a diameter of 4.0 mm and partial thread were used for fixation according to the size of the fracture block during the operation. Spacers can be used according to the intraoperative conditions. After the fluoroscopic position and depth of the C-arm machine were good during the operation, the incision was sutured layer by layer after a drainage tube was inserted (Figure 2). An illustration of surgical anatomy of PCL tibial avulsion fracture is shown in Figure 3. The knee joint of the affected limb was fixed at a flexion position of 30°–45° using a functional adjustable knee brace.

### Postoperative treatment and rehabilitation

Antibiotics were routinely used once within 24 h after operation to prevent infection. A functional adjustable knee brace was used to fix the knee joint of the affected limb at a flexion position of 30°–45°, and a cotton pad was used to prevent posterior displacement of the tibia. After returning to the ward, the patient was encouraged to start ankle pump exercise, quadriceps femoris contraction, and other functional exercises, and straight leg elevation training was performed 1 day after surgery. Passive knee flexion range of motion (0°–60°) was completed within 3 weeks after operation. At 4–6 weeks after surgery, the patient was in active flexion (0°–90°) in the prone position. After 8 weeks, normal knee movement can be restored, and the knee brace of the affected limb can be removed. During the first 4 weeks after surgery, toe contact or partial weight bearing on the leg was allowed, and physical therapy for knee mobility was initiated. Full weight bearing was permitted only after radiographs showed good evidence of bone healing. After 2–3 months, when sufficient strength, knee joint range of motion (KROM), and proprioceptive skills are restored, return to heavy strength or competitive physical activity is limited. The minimally invasive approach group could be exercised as early as the patient could tolerate. A regular monthly x-ray examination was performed to observe whether avulsion fracture of the affected limb reached the clinical healing standard. IKDC score, Lysholm score, and KROM score were recorded for the first time and each time after operation to evaluate the improvement of knee joint function and stability of the affected limb.

### Observation index

The Lachman test and posterior drawer test were observed after limb surgery. Patients were followed up at 4 weeks, 6 weeks, 2 months, 3 months, and every month thereafter, during which the affected limb was evaluated using the IKDC form and the Lysholm scale. The range of motion and residual relaxation of the knee were measured. All clinical evaluations were performed by two independent observers who were blinded to the surgical procedure. The IKDC score, Lysholm score, knee range of motion, and residual relaxation of the affected limb at 1 year after operation were analyzed to evaluate the recovery of the affected limb.

### Statistical methods

For data analysis, the statistical software Spss23.0 was used. Measurement data with a normal distribution were expressed as mean standard deviation, and the *t*-test was used to compare the Lysholm score, IKDC score, and residual relaxation between preoperative and postoperative patients. *P* < 0.05 indicates statistical significance between the two groups, which is used to assess the clinical efficacy of surgery.

## Results

All the operations were successfully completed, and the incision was healed at stage I. There were no complications such as incision infection, intra-articular infection, joint fibrosis, deep vein thrombosis, and fracture nonunion. The drawer test after Lachman test was negative, and the knee stability recovered well after operation. All 26 patients were followed up 18–24 months, with an average of 24.42± 5.01 months. The incision length was 3–6 cm, with an average of 4.04 ± 0.82 cm. The intraoperative blood loss was about 45–60 ml, with an average of 48.85 ± 5.88 ml. The operation time was 39–64 min, with an average of 52.46 ± 7.64 min. The postoperative hospital stay was 2–5 days, with an average of 2.73 ± 0.87 days. All fractures healed 8–12 weeks, with an average healing time of 9.46 ± 1.33 weeks (Table 1).

**Table 1:** Summary of general patient characteristics

Parameter	
Numbers of male: female patients	15:11
Age (years)	32.12 (22–55)
Interval from injury to surgery (day)	4.92 ± 1.32 (3–7)
Postoperative hospital stay	2.73 ± 0.87 (2–5)
Surgery time (min)	54.46 ± 7.64 (39–64)
Follow-up (months)	24.42 ± 5.01 (18–36)
Postoperative KROM (°)	137.08 ± 5.59 (129–148)
Fracture union time (week)	9.46 ± 1.33 (8–12)
Intraoperative blood (ml)	48.85 ± 5.88 (45–60)

KROM, knee joint range of motion.

The Lysholm score of the affected knee was 89–98 (mean 94.12 ± 2.49) at 1 year follow-up. The IKDC score was 87–95 with an average of 91.85 ± 2.19, and the KROM was 129°–148° with an average of 137.08 ± 5.59°, which was statistically significant compared with that before surgery (*P* < 0.05) (Table 2).

**Table 2:** Comparison of preoperative and postoperative knee function in minimally invasive approach.

Parameter	Preoperative	1 year postoperative	*T*-value	*P*-value
Lysholm	35.85 ± 1.22	94.12 ± 2.49	−53.402	0.000
IKDC	40.58 ± 4.41	91.85 ± 2.19	−128.760	0.000
Instability	10.73 ± 3.33	1.46 ± 0.65	14.300	0.000

IKDC, International Knee Joint Documentation Committee.

## Discussion

The aim of this study was to explore a new, minimally invasive, and safe surgical method for tibial PCL avulsion fracture that could fully expose the field of vision in a safer way without requiring large incisions, reduce the dissection and treatment of the surrounding muscle tissue, and avoid damage to the surrounding vascular and nerve systems.

The strength of the PCL is about twice that of the ACL, and it is considered to be the strongest ligament in the knee joint, which plays an important role in the stability of the joint (19). The incidence of PCL rupture is lower than that of other ligaments, which is related to its strong fibrous structure (20). PCL tibial avulsion fracture is a special form of PCL injury that is relatively rare compared with typical PCL tear (17, 21). The treatment of PCL tibial avulsion fracture is mainly divided into conservative treatment and surgical treatment. Zhao et al. believed that nonsurgical treatment can be successfully used for fractures with displacement less than 5 mm (22). Yoon et al. found that conservative treatment could achieve satisfactory results when the displacement of a simple PCL avulsion fracture was less than 6.7 mm (18). Although these conservative treatments have achieved good results, there are many complications in the late stages of the conservative treatment of displaced PCL tibial avulsion fractures, which are easy to lead to knee instability, severe mobility limitation, and knee degeneration. In order to prevent instability and further degenerative changes, early operation should be performed (23–25).

At present, the surgical options for PCL tibial avulsion fracture mainly include arthroscopic repair and open reduction and internal fixation. According to certain studies, the clinical efficacy of open approach and arthroscopic fixation of PCL tibial avulsion fractures is comparable (23, 26, 27). Since the use of arthroscopic surgery was first reported in 1995, with the progress of arthroscopic technology, arthroscopic treatment of PCL tibial avulsion fractures has been widely concerned (4, 28, 29). Although arthroscopic technology provides a minimally invasive technique and can be used in the same environment to handle any accompanying advantage of the meniscus, the synovial membrane and the surrounding ligament injury (14, 29–31), on the contrary, in addition to the requirements of technical and logical reasoning, it shows a higher rate of arthrofibrosis, longer operation time, high technical requirements, a long learning curve, the need for a specific device. In contrast to the open technique, it is not possible to see the avulsion fragment directly. In addition, arthroscopic reduction and fixation are more challenging than open surgery (32, 33).

Previous studies have reported a variety of surgical methods for tibial PCL avulsion fractures. Burks and Schaffer first described in 1990 a simplified retrogenicular inverted “L” approach that has become the standard open surgical approach for avulsion fractures of the PCL. The incision of this surgical approach is large and easy to damage the blood vessels and nerves (34). Nicandri et al. describe the “S” shape incision, this is a kind of improved after into the road, intraoperative do not need to remove gastrocnemius. However, this operation requires the separation and ligation of popliteal nerves, blood vessels, and muscles to find the fracture end, which increases the difficulty of the operation and postoperative complications (35).

This study's clinical results are comparable with those of recent studies that used different methods and techniques. The average operation time in this study was 54.46 ± 7.34 min, the average fracture union time was 9.46 ± 1.33 weeks, average Lysholm score was 94.12 ± 2.49, and average IKDC score was 91.85 ± 2.19. All patients' functional knee range of motion was restored, including full extension with a flexion of 137.08 ± 5.59° and an average residual relaxation of 1.46 ± 0.65 mm. Khalifa et al. described 31 cases involving a small set plate and plate fixation *via* an open “S” approach. Lysholm score was 93.4 ± 3.9. The knee flexion was 120.7°, which allowed for full extension. The incision was large, and there was significant vascular and nerve damage in the popliteal fossa (10). Bi et al. reported 15 cases of three-channel arthroscopic surgery involving autologous tendon transplantation, TightRope, and interference screw fixation. Lysholm's score was 94.25 ± 3.32. The residual relaxation was 1.08 ± 0.86 mm, and IKDC score was 91.13 ± 3.78. This arthroscopic surgery causes little trauma and can repair joint tissue damage. However, the operation is difficult and requires special instruments, which are difficult to obtain in primary care hospitals (36). Among the 36 patients described by Hao et al., 20 were treated with a self-made hook plate posterior medial inverted “L” approach and 16 with an EndoButton under arthroscopy. The inverted “L” approach took 57.80 ± 5.60 min to complete. Fracture union took 11.05 ± 2.21 weeks, Lysholm score was 95.50 ± 3.19, and the knee flexion was 134.80 ± 4.94°. The arthroscopic operation took 67.81 8.69 min, the fracture union time was 11.88 ± 2.25 weeks, Lysholm score was 95.19 ± 2.61, and the knee flexion was 131.44 ± 7.30°. The clinical efficacy of the traditional approach and arthroscopy were compared in this study. There were no significant differences in fracture healing time, Lysholm score, or knee flexion between the two groups at the last follow-up, but the operation time of the traditional approach group was significantly shorter than that of the arthroscopy group, and the difference was statistically significant. In this study, there was no significant difference in fracture healing time, Lysholm score, or knee flexion operation time when compared with the traditional inverted “L” approach. However, in our study, the surgical incision was smaller, the postoperative appearance was more attractive, the damage to the surrounding soft tissues was less severe, and the patients were able to exercise sooner (12). Gavaskar et al. described 22 patients whose surgical methods were similar to those used in this study, including the use of a small incision behind the knee. The difference was that Gavaskar et al. used a C-arm machine for fluoroscopic positioning prior to surgery to improve the accuracy of the incision position. The use of a 2-cm-wide Langenbeck retractor to separate the popliteal vascular and nerve bundles during surgery resulted in better visual field exposure and a shorter operation time (mean, 40 min), which is worth learning. However, the researchers separated the vascular and nerve bundles from the popliteal fossa, which could cause damage (37).

In this study, through a small transverse incision, the natural muscle gap between the medial head of the gastrocnemius muscle and the semitendinosus muscle was separated, and the vascular nerve bundle at the popliteal fossa was pulled to the lateral side together, thus avoiding the injury of the vascular nerve during operation. The operation can be performed under direct vision, which is beneficial to the reduction of the fracture block. In this study, the length of the incision and the degree of satisfaction with the scar after the operation are obviously better than those of a traditional operation. The operation time was not obviously prolonged (10), and the operation time may be further shortened for senior orthopedic surgeons as the operation progresses. Compared with arthroscopy, the operation time was significantly reduced (38). As with other surgical approaches, after operation, IKDC score, Lysholm score, knee joint mobility, and residual relaxation were significantly improved (31, 39).

In the treatment of PCL tibial avulsion fracture by minimally invasive small incision behind the knee, the following experiences are obtained in combination with literature and clinical practice: (1) for the displaced PCL tibial avulsion fracture, the operation should be performed as soon as possible, the limb swelling is light, the hierarchy is clear, and the reduction is easy. (2) The surgical incision should be 3–4 cm, which is too small for exposure, easy to cause tissue damage when pulling, and difficult to adjust the direction when inserting guide wire. (3) It is difficult to detect the meniscus, anterior cruciate ligament, and other structural injuries in this incision, so x-ray, CT, MRI, and other related examinations should be completed before operation. (4) Hollow screws were used for fixation, and three-dimensional CT reconstruction was performed as far as possible to determine the size and displacement direction of the avulsion bone block. Postoperatively, adjustable chuck knee brace was used for fixation. (5) When separating deep soft tissue, blunt separation with fingers was used to avoid injury to the popliteal vascular and nerve bundles, and the level and deep structure of separation could be sensed at the same time. (6) When inserting the guide wire, the operation should be strictly standardized, and the protective coat should be used to prevent tissue involvement and damage to the blood vessels and nerves; the direction of screw placement should be perpendicular to the fracture surface, so as to make the pressure between bone blocks. (7) The bone should be anatomically repositioned, and the PCL should be anatomically repositioned to prevent ligament relaxation. (8) Screw into the need to grasp the strength, enough; if the bone is broken, the tooth gasket can be used to transform the crushed bone into a whole for processing and increase the fixed holding force.

There are numerous flaws in this study, including a short patient follow-up period and a lack of clinical data on the long-term prognosis of this treatment regimen. The research sample is small, and the evidence level of the present study is too low to provide specific clinical treatment guidance. The lack of a control group makes it difficult to explain the technique's benefits and drawbacks; this incision makes it difficult to complete the exploration and repair of other knee structures. Because the size of the fracture fragment was not taken into account during fixation, some smaller bone pieces were unable to be fixed with cannulated lag screws. Smaller fracture pieces can be repaired and reduced using anchors (40), sutures (41, 42), steel wires (11), and specific plates (12, 43).

In addition, the procedure can be difficult for obese or muscular patients because the technique requires the surgeon to open the gastrocnemius muscle to expose the fractured end. If exposure is difficult, the incision may need to be widened or the traditional procedure changed.

## Conclusion

This study describes a technique that can be used to complete the internal fixation of tibial PCL avulsions. It uses a minimally invasive approach to the knee joint's posteromedial interval that does not involve separation of the popliteal nerve or blood vessels. In addition, the technique can be applied in source-constrained or smaller hospitals that are not equipped with arthroscopy technology.

## Data Availability

The original contributions presented in the study are included in the article/**Supplementary Material,** further inquiries can be directed to the corresponding author.
