# Corrigendum: Treatment of avulsion fracture of posterior cruciate ligament tibial insertion by minimally invasive approach in posterior medial knee

**DOI:** 10.3389/fsurg.2023.1173814

**Published:** 2023-03-27

**Authors:** Huihui Guo, Yao Zhao, Liang Gao, Chen Wang, Xianbo Shang, Haitao Fan, Wendan Cheng, Chang Liu

**Affiliations:** ^1^ Fuyang People's Hospital, Fuyang, China; ^2^ Department of Orthopedics, The Second Affifiliated Hospital of Anhui Medical University, Hefei, China; ^3^ Center for Clinical Medicine, Huatuo Institute of Medical Innovation (HTIMI), Berlin, Germany; ^4^ Anhui Medical University, Fuyang, China; ^5^ Anhui Armed Police General Hospital, Hefei, China

**Keywords:** minimally invasive, posterior cruciate ligament, avulsion fracture, clinical effects, technique

A Corrigendum on Treatment of avulsion fracture of posterior cruciate ligament tibial insertion by minimally invasive approach in posterior medial knee By Guo H, Zhao Y, Gao L, Wang C, Shang X, Fan H, Cheng W and Liu C. (2023) Front. Surg. 9:885669. doi: 10.3389/fsurg.2022.885669

**Incorrect Affiliation**

In the published article, there was an error in affiliation(s) [Huihui Guo]. Instead of “^1^Department of Orthopedics, The Second Affifiliated Hospital of Anhui Medical University, Hefei, China; ^2^Fuyang People’s Hospital, Fuyang, China.”, it should be “^1^Fuyang People’s Hospital, Fuyang, China; ^2^Department of Orthopedics, The Second Affifiliated Hospital of Anhui Medical University, Hefei, China”.

In the published article, there was an error in affiliation(s) [Liang Gao]. Instead of “^3^Center for Clinical Medicine, Hua Tuo Institute of Medical Innovation (HTIMI), Berlin, Germany”, it should be “^3^Center for Clinical Medicine, Huatuo Institute of Medical Innovation (HTIMI), Berlin, Germany”.

The authors apologize for this error and state that this does not change the scientific conclusions of the article in any way. The original article has been updated.

**Incorrect Funding**

In the published article, there was an error in the Funding statement. The corresponding Funding for this article was missing when the article was published. The correct Funding statement appears below.

**FUNDING**

[This study was supported by the Hefei independent innovation policy “Borrow-transfer-supplement” project (J2020Y07), Functional limb salvage of diabetic foot (2020byzd347), Masquelet technique combined with transverse bone transfer technique for the treatment of refractory Wagner III and IV diabetic (FY2021-027) and tibial periosteum lateral extension for Wagner III and IV diabetic foot in a multidisciplinary collaborative mode (2022xkj225). The funders had no role in study design, data collection and analysis, decision to publish, or preparation of the manuscript.]

The authors apologize for this error and state that this does not change the scientific conclusions of the article in any way. The original article has been updated.

**Text Correction**

In the published article, there was an error. [Retrospective analysis of 26 patients with PCL tibial avulsion fracture who met the screening criteria from January 2015 to January 2020 in the Department of Orthopaedics of the Second Affiliated Hospital of Anhui Medical University].

A correction has been made to **[Clinical data]**, *[General information],* [Third paragraph]. This sentence previously stated:

“[Retrospective analysis of 26 patients with PCL tibial avulsion fracture who met the screening criteria from January 2015 to January 2020 in the Department of Orthopaedics of the Second Affiliated Hospital of Anhui Medical University .]”

The corrected sentence appears below:

“[From January 2015 to January 2020, 26 cases of PCL tibial avulsion fracture were studied retrospectively at the orthopaedics departments of both Second Affiliated Hospital of Anhui Medical University and Anhui Armed Police General Hospital, Hefei, China.]”

In the published article, there was an error. [All patients were operated by the same orthopedic surgeon.].

A correction has been made to **[Clinical data]**, *[Surgical technique],* Sixth paragraph]. This sentence previously stated:

“[All patients were operated by the same orthopedic surgeon.]”

The corrected sentence appears below:

“[All of the patients were operated on by three senior orthopedic surgeons of the same team.]”

The authors apologize for this error and state that this does not change the scientific conclusions of the article in any way. The original article has been updated.

