# Supplementary material for: Treatment of avulsion fracture of posterior cruciate ligament tibial insertion by minimally invasive approach in posterior medial knee
Source: Front Surg. 2023 Jan 6;9:885669. doi: 10.3389/fsurg.2022.885669 (PMC9852621; doi:10.3389/fsurg.2022.885669)
Supplement: Supplementary file 1 [file Datasheet1.doc]

Clinical data of patients

| **Sex/age (years)** | **Duration of injury (days)** | **Postoperative hospital stay** | **Surgery time (min)** | **Intraoperative blood (ml)** | **Follow-up (months)** | **Fracture healing time（week）** |
| --- | --- | --- | --- | --- | --- | --- |
| **male/28** | **3** | **3** | **55** | **45** | **18** | **8** |
| **male/33** | **5** | **2** | **42** | **50** | **30** | **10** |
| **male/24** | **6** | **2** | **40** | **40** | **23** | **12** |
| **male/32** | **7** | **2** | **57** | **55** | **25** | **9** |
| **male/28** | **7** | **3** | **61** | **50** | **30** | **11** |
| **male/31** | **5** | **3** | **63** | **45** | **22** | **8** |
| **female/22** | **4** | **4** | **45** | **50** | **19** | **10** |
| **male/36** | **5** | **2** | **47** | **40** | **20** | **9** |
| **female/22** | **4** | **2** | **54** | **55** | **25** | **8** |
| **female/35** | **7** | **2** | **51** | **60** | **26** | **9** |
| **male/47** | **3** | **3** | **53** | **45** | **36** | **10** |
| **male/55** | **4** | **2** | **47** | **45** | **20** | **8** |
| **male/26** | **7** | **2** | **64** | **55** | **19** | **11** |
| **female/43** | **6** | **3** | **60** | **50** | **31** | **10** |
| **female/26** | **5** | **2** | **57** | **50** | **23** | **8** |
| **female/36** | **4** | **4** | **46** | **45** | **21** | **12** |
| **male/50** | **4** | **5** | **53** | **50** | **34** | **9** |
| **female/34** | **3** | **3** | **63** | **45** | **20** | **10** |
| **male/32** | **4** | **4** | **45** | **40** | **30** | **8** |
| **female/31** | **6** | **2** | **43** | **40** | **21** | **9** |
| **female/34** | **6** | **2** | **58** | **55** | **28** | **8** |
| **male/29** | **5** | **3** | **39** | **60** | **26** | **12** |
| **female/30** | **5** | **2** | **63** | **50** | **25** | **9** |
| **female/24** | **4** | **4** | **52** | **50** | **24** | **10** |
| **male/43** | **6** | **2** | **49** | **55** | **21** | **8** |
| **male/26** | **3** | **3** | **57** | **45** | **18** | **10** |

| **Preop IKDC**  **score** | **Postop IKDC score** | **Preop**  **Lysholm**  **score** | **Postop Lysholm**  **score** | **Postop ROM**  **(°)** | **Preop**  **Residual laxity**  **（mm）** | **Postop**  **Residual laxity**  **（mm）** |
| --- | --- | --- | --- | --- | --- | --- |
| 48 | 95 | 35 | 95 | 129 | 7 | 1 |
| 35 | 91 | 36 | 95 | 131 | 8 | 1 |
| 45 | 93 | 37 | 97 | 138 | 13 | 2 |
| 40 | 93 | 36 | 94 | 135 | 7 | 1 |
| 38 | 89 | 33 | 89 | 142 | 9 | 2 |
| 43 | 91 | 36 | 92 | 146 | 8 | 1 |
| 40 | 90 | 36 | 96 | 138 | 8 | 3 |
| 37 | 93 | 37 | 94 | 134 | 9 | 2 |
| 50 | 92 | 38 | 97 | 132 | 16 | 1 |
| 40 | 93 | 37 | 91 | 141 | 17 | 1 |
| 38 | 95 | 35 | 93 | 133 | 11 | 1 |
| 34 | 94 | 36 | 98 | 140 | 10 | 2 |
| 44 | 95 | 34 | 95 | 130 | 8 | 1 |
| 41 | 94 | 35 | 94 | 133 | 13 | 1 |
| 40 | 87 | 35 | 90 | 141 | 15 | 2 |
| 34 | 91 | 36 | 94 | 137 | 14 | 3 |
| 50 | 91 | 37 | 95 | 136 | 8 | 1 |
| 40 | 92 | 35 | 89 | 147 | 13 | 1 |
| 36 | 95 | 34 | 95 | 129 | 9 | 2 |
| 43 | 88 | 37 | 94 | 130 | 17 | 1 |
| 36 | 90 | 38 | 97 | 135 | 12 | 2 |
| 44 | 90 | 35 | 97 | 145 | 8 | 1 |
| 42 | 92 | 36 | 94 | 138 | 9 | 1 |
| 40 | 90 | 37 | 92 | 140 | 15 | 2 |
| 38 | 93 | 35 | 93 | 136 | 8 | 1 |
| 39 | 91 | 36 | 97 | 148 | 7 | 1 |
